# SmartCrop: knowledge base of molecular genetic mechanisms of rice and wheat adaptation to stress factors

**DOI:** 10.18699/vjgb-25-129

**Published:** 2025-12

**Authors:** P.S. Demenkov, T.V. Ivanisenko, M.A. Kleshchev, E.A. Antropova, I.V. Yatsyk, A.R. Volyanskaya, A.V. Adamovskaya, A.V. Maltseva, A.S. Venzel, H. Chao, M. Chen, V.A. Ivanisenko

**Affiliations:** Institute of Cytology and Genetics of the Siberian Branch of the Russian Academy of Sciences, Novosibirsk, Russia; Institute of Cytology and Genetics of the Siberian Branch of the Russian Academy of Sciences, Novosibirsk, Russia; Institute of Cytology and Genetics of the Siberian Branch of the Russian Academy of Sciences, Novosibirsk, Russia; Institute of Cytology and Genetics of the Siberian Branch of the Russian Academy of Sciences, Novosibirsk, Russia; Institute of Cytology and Genetics of the Siberian Branch of the Russian Academy of Sciences, Novosibirsk, Russia; Institute of Cytology and Genetics of the Siberian Branch of the Russian Academy of Sciences, Novosibirsk, Russia; Institute of Cytology and Genetics of the Siberian Branch of the Russian Academy of Sciences, Novosibirsk, Russia; Institute of Cytology and Genetics of the Siberian Branch of the Russian Academy of Sciences, Novosibirsk, Russia; Institute of Cytology and Genetics of the Siberian Branch of the Russian Academy of Sciences, Novosibirsk, Russia; Department of Bioinformatics, College of Life Sciences, Zhejiang University, Hangzhou, China; Department of Bioinformatics, College of Life Sciences, Zhejiang University, Hangzhou, China; Institute of Cytology and Genetics of the Siberian Branch of the Russian Academy of Sciences, Novosibirsk, Russia

**Keywords:** SmartCrop knowledge base, ANDSystem, text mining, artificial intelligent, molecular genetic mechanisms, rice, wheat, associative gene networks, abiotic stress, biotic stress, genotype–phenotype–environment interactions, omics technologies, long non-coding RNAs, marker-assisted selection, plant adaptation, stress resistance, база знаний SmartCrop, ANDSystem, извлечение знаний из текстов, искусственный интеллект, молекулярно-генетические механизмы, рис, пшеница, ассоциативные генные сети, абиотические стрессы, биотические стрессы, взаимодействия генотип–фенотип–среда, омиксные технологии, длинные некодирующие, РНК, маркер-опосредованная селекция, адаптация растений, стрессоустойчивость

## Abstract

The study of molecular genetic mechanisms of plant responses to specific growth conditions and stress factors is a central focus of scientific research aimed at developing new valuable crop varieties, particularly rice and wheat. These factors include abiotic stresses (high or low temperatures, drought, salinity, soil metal contamination), biotic stresses (pathogens, pests), as well as plant responses to regulatory factors (fertilizers, hormones, elicitors, and other compounds). Modern research in plant genetics is based on the understanding that the formation of any phenotypic characteristics (molecular genetic, biochemical, physiological, morphological, etc.) is controlled by gene networks – groups of coordinately functioning genes interacting through their products (RNA, proteins, and metabolites). Previously, we developed the ANDSystem intelligent technology designed to extract knowledge from scientific publication texts for the reconstruction of gene networks in biology and biomedicine. In this work, using an adapted version of ANDSystem for plants, we created the SmartCrop knowledge base designed to address challenges related to studying molecular genetic mechanisms of genotype-phenotype-environment interactions for agriculturally valuable rice and wheat crops. SmartCrop is designed to assist researchers in solving tasks such as interpreting omics technology results (establishing connections between gene sets and biological processes, phenotypic traits, etc.); reconstructing gene networks describing relationships between molecular genetic objects and concepts in breeding, phenomics, seed production, phytopathology, diagnostics, protective agents, etc.; identifying regulatory and signaling pathways of plant responses to specific growth conditions and biotic and abiotic stresses; predicting candidate genes for genotyping; searching for markers for marker-assisted selection; and identifying potential targets for substances (including external factors) affecting plants to ensure timely and uniform germination, better vegetative growth, efficient nutrient uptake, and improved stress resistance.

## Abstract (ru)

Изучение молекулярно-генетических механизмов реакций растений на специфические условия роста и стрессовые факторы – одно из приоритетных направлений исследований, нацеленных на создание новых сортов сельскохозяйственных культур, в частности риса и пшеницы. К числу таких факторов относятся абиотические стрессы (высокие или низкие температуры, засуха, засоление, загрязнение почвы металлами), биотические стрессы (патогены, вредители), а также реакции растений на регуляторные факторы (удобрения, гормоны, элиситоры и другие соединения). Современные исследования в области генетики растений основаны на понимании того, что формирование любых фенотипических характеристик (молекулярно-генетических, биохимических, физиологических, морфологических и др.) контролируется генными сетями – группами согласованно функционирующих генов, взаимодействующих через свои продукты (РНК, белки и метаболиты). Ранее с целью реконструкции генных сетей, значимых для биологии и биомедицины, нами была разработана интеллектуальная компьютерная система ANDSystem, предназначенная для автоматизированного извлечения знаний из текстов научных публикаций и баз данных. В настоящей работе, используя адаптированную версию ANDSystem для растений, мы создали базу знаний SmartCrop для решения задач, связанных с изучением молекулярно-генетических механизмов взаимодействий «генотип–фенотип–среда» для сельскохозяйственно ценных культур риса и пшеницы. SmartCrop предназначена для помощи исследователям в решении таких задач, как интерпретация результатов омиксных экспериментов на растениях: установление связей между наборами генов и биологическими процессами, фенотипическими признаками и др.; реконструкция генных сетей, описывающих отношения между молекулярно-генетическими объектами и понятиями в селекции, феномике, семеноводстве, фитопатологии; выявление регуляторных и сигнальных путей, ответных реакций растений на специфические условия роста и биотические и абиотические стрессы; прогнозирование генов-кандидатов для генотипирования; поиск маркеров для маркер-опосредованной селекции; выявление потенциальных мишеней (генов и белков) для субстанций, влияющих на растения (контролирующих процессы прорастания семян, вегетативного роста, эффективного поглощения питательных веществ и улучшения устойчивости к стрессовым факторам).

## Introduction

Rice (Oryza sativa L.) and wheat (Triticum aestivum L.) are among the most important agricultural crops, ensuring food security for a significant portion of the world’s population. Both crops are well known for their high nutritional, industrial, and fodder value (Shewry, Hey, 2015). Under current conditions, the production of these crops faces serious challenges. Extreme weather events, adverse climate change, plant diseases, and pests lead to substantial yield losses (Lesk et al., 2016). Overcoming these difficulties is impossible without studying the molecular genetic mechanisms underlying plant resistance to unfavorable biotic and abiotic factors, which requires the analysis of complex systems that include intricate signaling, regulatory, transport, and metabolic pathways (Mittler, 2006; Nykiel et al., 2023).

An effective tool for studying such mechanisms is gene networks, which control molecular genetic processes that determine the formation of phenotypic traits and the functioning of biological processes, including plant stress responses.

The modern concept of gene networks encompasses not only molecular components (RNAs, genes, proteins, and metabolites) but also a wide range of heterogeneous entities, including diseases, biological processes, and environmental factors. This type of gene network is known as an associative gene network (Ivanisenko V.A. et al., 2015). Structurally, such networks represent a knowledge graph integrating information about interactions among diverse objects involved in the functioning of molecular genetic systems or influencing them. In agrobiology and crop science, gene network analysis is successfully used to study economically important traits such as resistance to diseases and pests, tolerance to abiotic stress factors, and yield (Virlouvet et al., 2018; Chen et al., 2020).

The reconstruction of plant gene networks is a complex task that requires processing massive amounts of data and integrating fragmented information from scientific publications, including data on regulatory, transport, and catalytic processes, as well as relationships between genetic features, phenotypic manifestations, and environmental factors. To extract such knowledge, text-mining methods are applied, based both on classical computational approaches (dictionary-based methods, syntactic and linguistic rules and patterns, statistically significant co-occurrence, etc.) and on machine-learning techniques (Ivanisenko T.V. et al., 2014; Shrestha et al., 2024; Zhang et al., 2024).

Machine-learning algorithms used for constructing and analyzing gene networks can be divided into the following categories: supervised learning, unsupervised learning, semi-supervised learning, and hybrid approaches. Supervised learning methods rely on pre-annotated data to build predictive models, for example, to identify key regulators or to predict functional interactions between plant genes (Ni et al., 2016). Unsupervised learning enables the discovery of hidden patterns in large datasets, which is important, for instance, when clustering genes based on expression similarity or identifying gene-network modules. Semi-supervised learning combines the strengths of both approaches, using both labeled and unlabeled data, which is particularly relevant when the amount of well-annotated data is limited (Yan, Wang, 2022). Hybrid approaches integrate various machine-learning methods as well as traditional bioinformatics techniques, allowing them to effectively compensate for the limitations each approach may have when used alone (Guindani et al., 2024; Ivanisenko T.V. et al., 2024). For example, combining dictionary-based named-entity recognition in texts with machine-learning methods improves the accuracy of entity identification (Do et al., 2018; Ivanisenko T.V. et al., 2020).

In recent years, deep machine learning has achieved significant advances through the introduction of the transformer architecture and attention mechanisms, which have enabled substantial progress in natural language processing and the analysis of biological sequences (Vaswani et al., 2017). The analysis of gene networks has also seen considerable development with the application of graph neural networks, among which the GraphSAGE architecture (Hamilton et al., 2017) enables efficient training on large heterogeneous graphs by aggregating features from neighboring nodes. A promising direction is the use of large language models, such as Gemma-2-9b-it (Gemma Team, Google DeepMind, 2024), which provide high-quality semantic analysis of scientific texts and validation of extracted interactions

A number of specialized resources have been developed for the reconstruction and analysis of plant gene networks. These include PlantRegMap (Tian et al., 2020), designed for analyzing transcription factor regulatory interactions; STRING (Szklarczyk et al., 2021), which enables the exploration of protein–protein interactions; the KEGG PLANT platform (Kanehisa, 2013), which integrates information on metabolic pathways across various plant species; and the Plant Reactome resource (Naithani et al., 2020), containing detailed data on signaling and metabolic pathways in model plant organisms. For visualization and analysis of gene networks, the Cytoscape software environment (Otasek et al., 2019) is widely used, offering an extensive set of plugins for working with biological data. The ncPlantDB database provides comprehensive information for analyzing regulatory networks, including data on cell-type specific expression of noncoding RNAs and their interactions (Cheng et al., 2024; Liu et al., 2025). The integration of such omics resources forms an effective platform for reconstructing gene networks of agricultural crops (Chao et al., 2023).

Earlier, we developed the ANDSystem cognitive software information platform (Ivanisenko V.A. et al., 2015, 2019; Ivanisenko T.V., 2020, 2022) designed for the full knowledge-engineering cycle in the biomedical domain. The system’s knowledge base contains more than 50 million interactions for various organisms

In the field of plant biology, ANDSystem has been used to create a knowledge base on the genetics of Solanum tuberosum (Saik et al., 2017; Ivanisenko T.V. et al., 2018; Demenkov et al., 2019), to reconstruct and analyze the regulatory gene network controlling cell wall functions in Arabidopsis thaliana leaves under water deficit (Volyanskaya et al., 2023), and to develop a method for prioritizing biological processes based on the reconstruction and analysis of associative gene networks (Demenkov et al., 2021).

The application of the ANDSystem automated reconstruction of associative gene networks to analyze microRNAmediated regulation of bread wheat (Triticum aestivum L.) adaptation to water deficit made it possible to propose new candidate microRNAs (MIR7757, MIR9653a, MIR9670, MIR9672b) of interest for further experimental studies of plant adaptation mechanisms under insufficient moisture (Kleshchev et al., 2024).

In another study (Antropova et al., 2024), ANDSystem was used to reconstruct the molecular genetic network of rice (Oryza sativa) responses to Rhizoctonia solani infection under nitrogen excess, which revealed three potential mechanisms explaining reduced plant resistance to the pathogen. Key regulatory pathways were identified: an OsGSK2-mediated cascade, the OsMYB44–OsWRKY6–OsPR1 signaling pathway, and a pathway involving SOG1, Rad51, and the PR1/PR2 genes. In addition, markers promising for breeding were identified: 7 genes regulating a broad range of stress responses and 11 genes that modulate the immune system. Additional analysis of noncoding RNAs (Antropova et al., 2024) identified 30 microRNAs targeting genes within the reconstructed gene network. For two of them (Osa-miR396 and Osa-miR7695), approximately 7,400 unique long noncoding RNAs with differing co-expression indices were found, which may indicate a complex architecture of posttranscriptional regulation under nitrogen stress.

The aim of the present work was to adapt ANDSystem to create the SmartCrop knowledge base, integrating data on molecular genetic mechanisms and associative gene networks of stress responses in rice and wheat based on intelligent analysis of scientific publications and curated factual databases. This work included the development of a domain ontology and the optimization of intelligent knowledge-extraction methods from scientific texts using semantic–linguistic patterns and pretrained large language models. The SmartCrop ontology is represented by a set of interconnected dictionaries describing: molecular genetic entities (genes, proteins, metabolites, microRNAs), biological processes, phenotypic traits and diseases, pathogens, genetic biomarkers, markers of resistance to crop protection products, molecular targets of chemical crop protection agents, biotic and abiotic factors, crop protection products, as well as cultivars with their economically valuable and consumer traits.

As a result of automated analysis of scientific publications, the SmartCrop knowledge base was formed, integrating more than 10 million interactions among the entities defined in the ontology

## Materials and methods

Information resources used in the development of SmartCrop. To create the SmartCrop knowledge base, we used the ANDSystem software information platform (Ivanisenko V.A. et al., 2015, 2019; Ivanisenko T.V., 2020, 2022) and its information and bioinformatics technologies

Customization of ANDSystem methods for the subject domain was carried out using an ontology that included specialized dictionaries of entities and a description of the types of their interactions. The main sources of genetic and genomic information for constructing the dictionaries were: the NCBI Gene database (https://www.ncbi.nlm.nih. gov/gene), the rice-specific database Oryzabase (https:// shigen.nig.ac.jp/rice/oryzabase), the microRNA database miRBase (https://www.mirbase.org), the long noncoding RNA co-expression database ncPlantDB (https://bis.zju. edu.cn/ncPlantDB/ ), the single nucleotide polymorphism database dbSNP (https://www.ncbi.nlm.nih.gov/snp), and the database on cereal crops GrainGenes (https://wheat. pw.usda.gov/GG3)

To standardize terminology, we used the following ontologies: Gene Ontology (http://geneontology.org), Crop Ontology for wheat and rice (https://cropontology.org), as well as the genetic resources collection of VIR (https:// www.vir.nw.ru).

Chemical compounds and metabolites were annotated using the ChEBI database (https://www.ebi.ac.uk/chebi). Information on herbicide resistance was obtained from the International Herbicide-Resistant Weed Database (http://www.weedscience.org), and data on pesticides were taken from the EU Pesticide Database (https://food. ec.europa.eu/plants/pesticides/eu-pesticides-database_en). For knowledge extraction from texts, we used ANDSystem’s semantic-linguistic templates, as well as newly developed templates tailored to the specifics of the subject domain. In addition, artificial intelligence methods were applied for knowledge extraction, including GraphSAGE graph neural networks (Hamilton et al., 2017) and the large language model Gemma-2-9b-it (Gemma Team, Google DeepMind, 2024).

Evaluation of accuracy. To assess the quality of namedentity annotation in the text, the F1-score was used, which is the harmonic mean of precision (Precision) and recall (Recall):

F1 = 2·(Precision × Recall)/(Precision + Recall), Precision = TP/(TP + FP), Recall = TP/(TP + FN), where TP – are true positives, FP – are false positives, and FN – are false negatives.

## Results

**Fig. 1.**
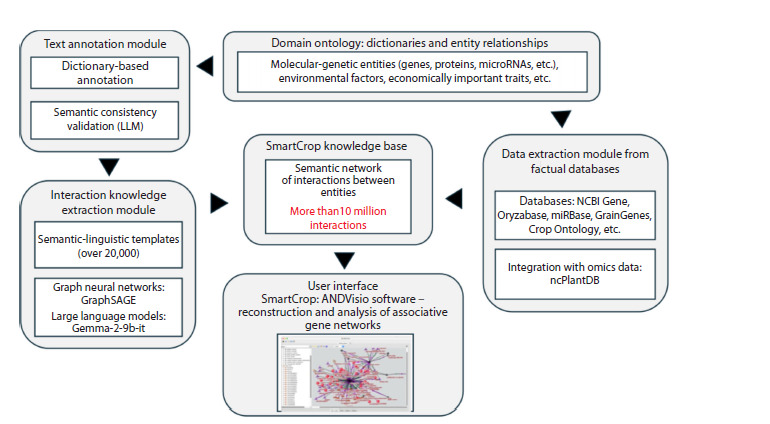
Schematic representation of the architecture of the SmartCrop software information system.

A schematic representation of the main components of the SmartCrop software-information system is shown in Figure 1.

**SmartCrop domain-specific ontology module**

The development of a domain-specific ontology was a key stage in building SmartCrop. The domain-oriented ontology defines a conceptual model of the problem area and includes dictionaries of entities and types of their interactions. Based on these dictionaries, information about interactions between specific entities is extracted from texts and factual databases. The current version of the SmartCrop ontology contains 15 dictionaries of different entity types (Table 1), compiled by extracting entity names from specialized databases and existing ontologies.

**Table 1.**
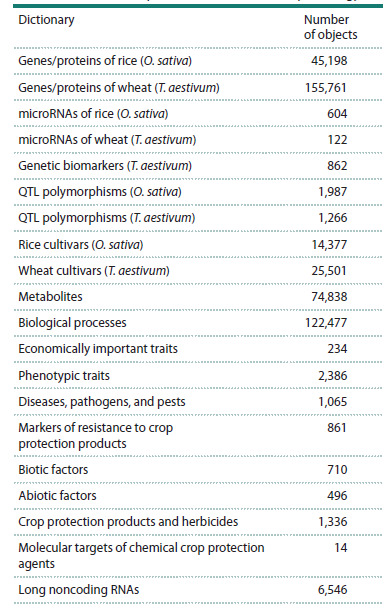
Dictionaries represented in the SmartCrop ontology

Interaction types. In the SmartCrop system, 16 types of relationships between ontology entities are defined. All interactions in the system are directional and can be divided into several main groups. Physical interactions include processes of forming both short-lived molecular complexes and stable associations between proteins and metabolites.

Chemical interactions comprise catalytic reactions of the substrate–enzyme–product type, protein proteolysis, as well as various post-translational protein modifications such as phosphorylation and glycosylation

A distinct group is formed by regulatory interactions, which encompass the regulation of gene expression by transcription factors, modulation of protein activity and function, control of protein and metabolite transport, as well as regulation of protein stability and degradation. An important feature is that regulatory interactions also define relationships between molecular genetic entities, biological processes, and phenotypic traits. Each regulatory event may be characterized by an enhancing or attenuating effect on the corresponding process.

Expression and co-expression of genes are distinguished separately. The products of gene expression are proteins and noncoding RNAs. Co-expression is the simultaneous expression of genes driven by shared regulatory mechanisms under changing cellular conditions. Additionally, the system accounts for associative links, which include unclassified interactions between various ontology entities.

**Text-annotation module based on ontology entities**

Recognition of molecular genetic entities in scientific texts is a challenging task due to the specific nature of their nomenclature. Our experience with ANDSystem shows that a substantial portion of errors in automatic reconstruction of associative gene networks is associated with inaccurate identification of named entities (Ivanisenko T.V. et al., 2022). The causes of such errors include the use of abbreviations by authors, semantic ambiguity of terms, and various linguistic features of scientific texts. In publications, standard names of entities are often modified, punctuation and word order are altered, grammatical forms vary, abbreviations are used, or technical typos are introduced (Pearson, 2001; Krallinger et al., 2015; Islamaj et al., 2021).

To improve recognition accuracy, we developed a twostage process: 1) initial matching of names to the ontology dictionary and 2) subsequent verification of whether each annotated entity name corresponds to its type, based on contextual document analysis using neural networks.

The verification process is implemented as follows: a language model converts the context (about 400 words) containing the analyzed entity, which is replaced with a special mask tag, into a vector representation. Based on this representation, a neural network performs binary classification, determining whether the contextual environment of the term is consistent with its typical usage

For entities from the ANDSystem ontology (genes, proteins, metabolites, etc.), classification accuracy was reported in a previously published paper (Ivanisenko T.V. et al., 2022). For the new SmartCrop dictionaries, manual expert evaluation of annotation quality was carried out (Table 2) based on the analysis of 1,000 randomly selected documents from the PubMed and PubMed Central databases

**Table 2.**
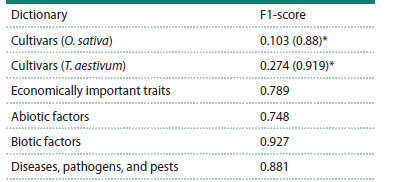
Accuracy assessment of entity name annotation for the new dictionaries

The evaluation results demonstrated high annotation accuracy for most dictionaries, with the exception of rice and wheat cultivar names. The identification of plant cultivar names is a well-known complex task, determined by several factors, including substantial overlap of terms with common vocabulary and anthroponyms, as well as the lack of a unified standard in the nomenclature of new cultivars (Do et al., 2018; D’Souza, 2024).

To address this problem, a specialized language model was trained, focused on the task of contextual term classification. The training was carried out in accordance with the methodology previously described in our work on improving the accuracy of identifying eight types of molecular genetic entities, including proteins, genes, metabolites, and cellular components (Ivanisenko T.V. et al., 2022). Integration of the developed model made it possible to substantially increase the recognition accuracy (F1-score) of cultivar names to 0.88 for rice and 0.919 for wheat.

**Knowledge extraction module**

The knowledge extraction module for scientific texts implements three main stages: 1) primary knowledge extraction using semantic-linguistic templates; 2) reconstruction of the initial semantic network; 3) its extension using graph neural networks and large language models. Additionally, to further expand the semantic network, a data extraction module for factual databases containing structured information is used, which makes it possible to obtain additional information about interactions between entities.

Semantic-linguistic templates are structured records containing metadata about the types of entities and the nature of their interactions. They include two main components: 1) syntactic relations that describe the order of entities and keywords in a sentence using regular expressions, and 2) semantic relations that define the type of interaction between entities. Regular expressions are used to search for patterns in the arrangement of entity names in annotated text sentences. When a match is found, specific entity names from the text are mapped to the template identifiers.

For each interaction type, specialized groups of templates with unique syntactic rules were developed. The knowledge base contains more than 18,000 ANDSystem templates for interaction types represented in both the ANDSystem and SmartCrop ontologies, as well as more than 3,000 templates specifically designed for the rice and wheat ontologies. The effectiveness of the template-based interaction extraction method was demonstrated during the development of ANDSystem (Ivanisenko V.A. et al., 2015).

Application of graph neural networks and large language models. At the second stage, based on the knowledge extracted using templates, a primary knowledge graph (semantic network) was constructed and used to train a graph neural network. After training, the network was used to predict missing edges in the knowledge graph. At the third stage, large language models were applied to validate these predictions by analyzing scientific texts in which the annotated entities with the predicted interactions co-occur (Ivanisenko T.V. et al., 2024).

**Integration with omics data**

Noncoding RNAs (ncRNAs) represent a broad and functionally diverse class of RNA molecules that are not translated into proteins but perform key regulatory functions in the cell. Long noncoding RNAs (lncRNAs) are of particular inte- rest, as they participate in the regulation of gene expression at multiple levels – from modulating mRNA stability and translation to being involved in complex signaling cascades (Statello et al., 2021; Supriya et al., 2024).

A well-known specialized resource on ncRNA co-expression in plants, including rice lncRNAs, is the ncPlantDB database (https://bis.zju.edu.cn/ncPlantDB/). It provides information on tissue-specific ncRNA expression at the single-cell level and their putative interactions, obtained using modern single-cell transcriptomics methods (Cheng et al., 2024; Liu et al., 2025). Integration of SmartCrop with ncPlantDB made it possible to use ncRNA co-expression data, including their relationships with microRNAs, to enrich the reconstructed gene networks.

**Module for gene network analysis and visualization**

**Fig. 2.**
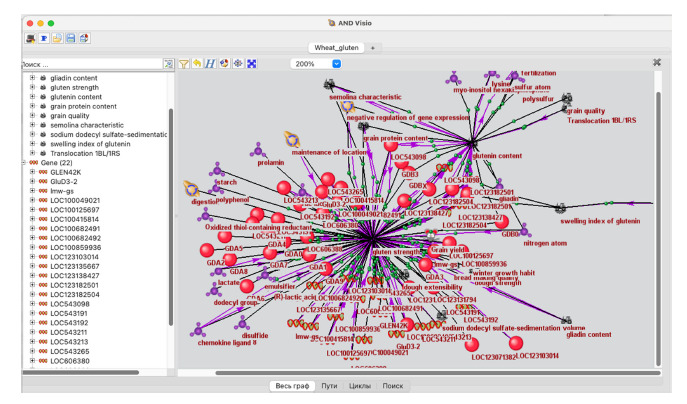
Screenshot of the ANDVisio program interface.

As the graphical user interface of SmartCrop, intended for the reconstruction and analysis of gene networks based on information from the SmartCrop knowledge base, the ANDVisio software is used (Fig. 2).

The ANDVisio program (Demenkov et al., 2012) was originally developed as a component of the ANDSystem platform and was later adapted for integration with Smart- Crop. It provides researchers with a wide range of tools for structural and functional analysis of gene networks, including:

• multiple graph layout algorithms; • a multi-parameter filtering system; • mechanisms for pathways and cycles finding; • tools for calculating node centrality measures; • tools for assessing the enrichment of biological processes with network genes; • additional methods of network analysis

**SmartCrop knowledge base**

The system’s knowledge base is implemented as a semantic network (knowledge graph) that integrates data extracted both from scientific publications and from factual databases. In this graph structure, nodes correspond to entities of the domain ontology, and edges represent various types of interactions between them

The knowledge base was populated through systematic analysis of the scientific literature, including abstracts from PubMed and full-text articles from the open-access resource PubMed Central. The time span of the analyzed publications covered the period from 1970 to 2024, with the main selection criterion being the presence of references to wheat or rice. Detailed statistics on the number of recorded interactions in the SmartCrop knowledge base are presented in Table 3.

**Table 3.**
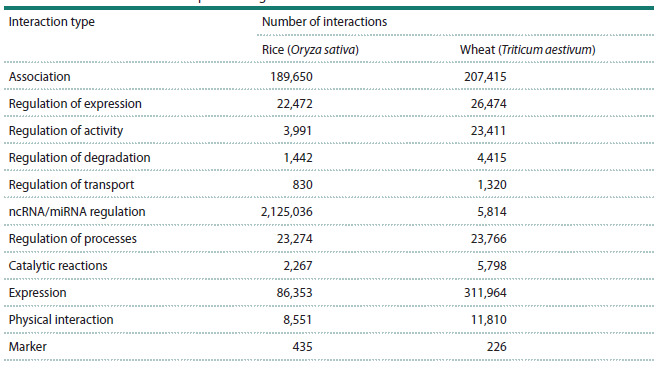
Statistics of the SmartCrop knowledge base on interactions between entities for wheat and rice

## Discussion

To demonstrate the capabilities of SmartCrop, we consider two use cases: analysis of experimental omics data and experiment planning.

**Analysis of experimental omics data**

As an example of omics data interpretation, we performed functional annotation of differentially expressed genes (DEGs) in bread wheat under salt stress. For the analysis, we used a set of 5,829 DEGs obtained from the NCBI GEO database (GSE225565, Alyahya, Taybi, 2023) for root tissues of bread wheat (Triticum aestivum L., cultivar Saudi) in response to salinization

The results of the overrepresentation analysis of Smart- Crop entities (biological processes, phenotypic traits, agronomically important traits, pathogens) for this DEG set and their protein products are presented in Supplementary Table S11. In total, significant overrepresentation (p-value < 0.05, Bonferroni-corrected) was found for 217 terms describing biological processes (entity type Pathway), 50 phenotypic traits (Phenotype), 9 agronomically important traits (Agrophenotype), and 38 pathogenic species. The list of entities belonging to the five groups of the most statistically significant characteristics is given in Table 4.

Supplementary Materials are available in the online version of the paper: https://vavilovj-icg.ru/download/pict-2025-29/appx46.xls


**Table 4.**
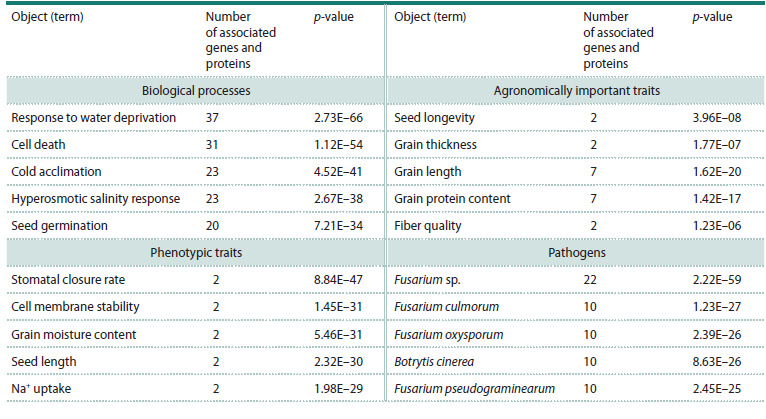
Bread-wheat characteristics significantly associated with the DEG set and their protein products under salt stress, identified using the SmartCrop system

Analysis of the overrepresented biological processes showed that the DEG set under study is associated not only with the response to salt stress, but also with the response to water deficit. This reflects plant adaptation mechanisms to the state of so-called “physiological drought”, which arises when effective water uptake becomes impossible due to high osmotic pressure of the surrounding environment. Among such adaptations is stomatal closure, mediated by a rapid increase in abscisic acid levels (Verma et al., 2016; Zhao et al., 2021). Accordingly, the significantly overrepresented entities included both the phenotypic trait “stomatal closure rate” (Table 4) and signaling pathways associated with abscisic acid (Table S1), which confirms their important role in the response to salinity

It should be noted that an important advantage of the SmartCrop knowledge base, compared with widely used resources for gene functional annotation (DAVID, Gene Ontology, ShinyGO, etc.), is the ability to analyze relationships between genes/proteins and not only biological processes, molecular functions, cellular components, and KEGG pathways, but also a broad spectrum of abiotic and biotic environmental factors, phenotypic traits, agronomically important properties, and pathogens. This integration makes it possible to assess overrepresentation for different types of entities in the gene set under study, substantially expanding the capabilities of functional annotation and enabling the identification of genes with pleiotropic effects. The latter is particularly important for marker-assisted selection, since selection based on a single target phenotypic trait or genetic marker may simultaneously affect several other, non-target traits.

In particular, the results of functional annotation of DEGs in bread wheat under salt stress showed their association not only with responses to salinity and water deficit, but also with seed germination and with agronomically important traits reflecting grain quality (Table 4). For example, aquaporins (encoded by genes LOC543267, LOC100037645, LOC123093445, and others) provide selective transport of water molecules, participate in maintaining cellular ion balance and in regulating water–salt homeostasis under elevated salinity (Ayadi et al., 2019), and also facili- tate the movement of water and solutes within seeds, which plays a key role in the germination process (Hoai et al., 2020).

The functionality of SmartCrop is not limited to overrepresentation analysis. The system also makes it possible to reconstruct associative networks of proteins and genes significantly associated with overrepresented entities and to search for their regulators. This provides a deeper understanding of the molecular mechanisms underlying these relationships and helps to reveal their specificity under experimental conditions.

**Fig. 3.**
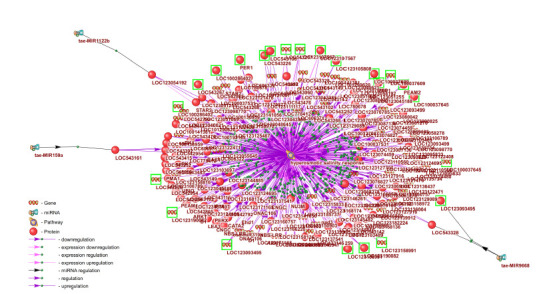
Genes, proteins, and microRNAs involved in regulating the response of bread wheat to hyperosmotic stress Genes differentially expressed in bread-wheat roots in response to soil salinization, as well as their protein products, are highlighted with a green frame

As an example, a gene network regulating plant tolerance to hyperosmotic stress (GO:0042538 hyperosmotic salinity response) was reconstructed (Fig. 3). According to SmartCrop, the wheat response to hyperosmotic stress involves 95 genes and 119 proteins, including aquaporins and sodium/hydrogen exchangers, which play a key role in regulating intracellular pH, water balance, and sodium-ion homeostasis (Gupta et al., 2021). Excess sodium ions entering from the environment are removed from the cytoplasm into the apoplast and vacuoles in exchange for hydrogen ions via transmembrane Na+/H+ exchangers (Zhao et al., 2021).

The network also includes peroxidases and catalases involved in antioxidant defense under abiotic stress; transcription factors of the MYB and WRKY families; dehydrins (LOC123125487, LOC100141381, and others); cold-shock proteins (LOC123080042, LOC543252, LOC542792); as well as DELLA proteins, which, by suppressing the gibberellin signaling pathway and interacting with jasmonic acid signaling, increase plant tolerance to abiotic stress, including salinity (Colebrook et al., 2014). In addition, the network contains calcium-dependent protein kinases – key components of calcium signaling cascades activated under abiotic stress

In addition, according to SmartCrop, the regulation of the response to hyperosmotic stress involves the microRNA tae- MIR159a, which regulates the expression of the transcription factor TaMyb3 (LOC543161), as well as tae-MIR1122b and tae-MIR9668, the targets of which are the aquaporins LOC123054192 and LOC123093495, respectively.

Of the full set of genes involved in the regulation of the hyperosmotic stress response, only nine showed differential expression in bread-wheat root tissues under experimental salt stress in the study (Alyahya, Taybi, 2023). This list includes genes encoding aquaporins, peroxidases, catalases, and the serine/threonine protein kinase CTR1 (LOC100286402). Thus, under the experimental conditions described by (Alyahya, Taybi, 2023), signaling pathways associated primarily with antioxidant defense were activated

**Fig. 4.**
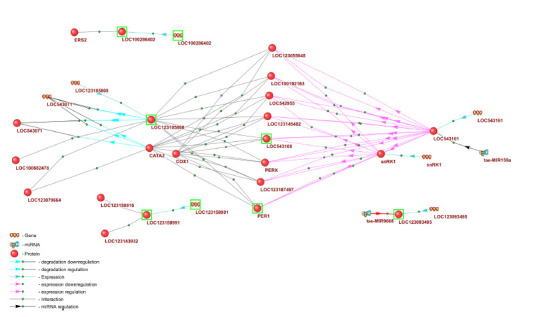
Gene network regulating the response of bread wheat to salt stress. Genes differentially expressed in bread-wheat roots in response to soil salinization, as well as their protein products, are highlighted with a green frame.

The associative network reconstructed in SmartCrop includes these nine DEGs and their protein products, regulatory proteins, as well as two microRNAs: tae- MIR159a, which regulates expression of the transcription factor TaMyb3 (LOC543161), and tae-MIR9668, targeting the aquaporin LOC123093495. This network is shown in Figure 4.

It is interesting to note that the transcription factor TaMyb3 (LOC543161), which is a target of the microRNA tae-MIR159a, in turn acts as a negative regulator of the expression of several genes encoding peroxidases. Suppression of the expression of these enzymes leads to increased accumulation of hydrogen peroxide in tissues and, consequently, to reduced plant tolerance to salinity (Wei et al., 2021). Thus, in this case a “cassette-cascade” regulatory principle involving microRNAs is implemented, in which a microRNA controls the expression of its target transcription factor, and the latter regulates an entire set of genes involved in the response to abiotic stress (Kleshchev et al., 2024).

Transcription factors of the MYB family are well known as regulators of responses to various abiotic stresses, including salinity (Kong et al., 2021; Wang S. et al., 2021). In particular, they participate in the regulation of flavonoid biosynthesis – metabolites required for protecting cells from oxidative stress (Wang X. et al., 2021).

**Application of SmartCrop to experimental design**

search for promising genes and phenotypic markers for subsequent marker-assisted selection and genome editing aimed at increasing rice (Oryza sativa L.) tolerance to soil salinity.

**Fig. 5.**
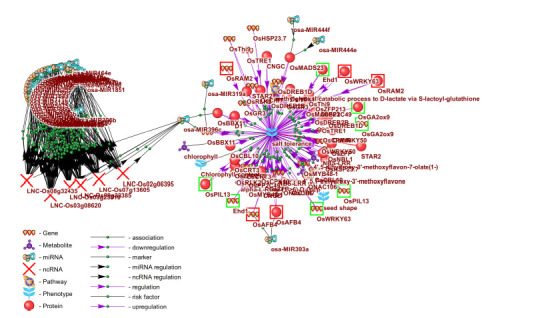
Associative gene network illustrating the involvement of genes, proteins, microRNAs, and long noncoding RNAs in the regulation of rice (Oryza sativa L.) tolerance to salinity and their potential role as phenotypic markers Genes in rice and the proteins they encode that positively regulate both salt tolerance and other agronomically important traits are outlined in green. Genes in rice and the proteins they encode that enhance salt tolerance but suppress other agronomically important traits are outlined in red.

According to SmartCrop, the following traits can serve as markers of salinity tolerance: chlorophyll content, seed shape, and the content of the metabolites 3′-methoxyapigenin and 5,7,4′-trihydroxy-3′-methoxyflavone. According to the SmartCrop knowledge base, rice tolerance to salinity is regulated by 30 genes and their corresponding 30 protein products (Fig. 5). In addition to genes, this regulation involves the microRNAs osa-MIR444f and osa-MIR444e, which target the transcription factor OsMADS23, as well as osa-MIR444e, targeting the auxin receptor OsABF4. The transcription factor OsBBX11, a known regulator of salinity tolerance (Lei et al., 2023), is targeted by the microRNAs osa-MIR319a and osa-MIR396c.

Long noncoding RNAs (lncRNAs) are molecules longer than 200 nucleotides that regulate gene expression at the transcriptional, post-transcriptional, and epigenetic levels, thereby modulating plant responses to various abiotic and biotic factors, including salinity (Sun X. et al., 2018). lncRNAs can interact with DNA (chromatin, promoters, and enhancers), proteins, mRNAs, and microRNAs. One important mechanism of their action is binding to microRNAs, which prevents the latter from acting on their targets and thus turns lncRNAs into key regulators of microRNA activity (Saha et al., 2025).

According to SmartCrop, the microRNA osa-MIR396c interacts with 508 long noncoding RNAs, six of which (LNC-Os02g06395, LNC-Os03g08620, LNC-Os03g25810, LNC-Os07g13605, LNC-Os08g32435, LNC-Os09g33385) are co-expressed not only with osa-MIR396c but also with 42 other rice microRNAs (Fig. 5). This indicates their potential role as key players in the regulation of rice tolerance to abiotic stresses, including salinity.

Of the 30 genes that regulate salinity tolerance, six (OsPIL13, OsNBL1, OsABF4, OsCPK10, OsCRT3, OsBBX11) control chlorophyll content. The remaining 24 genes have not previously been associated with known markers of rice salinity tolerance and therefore represent promising candidates for the discovery of new genetic markers of this trait

It should be particularly emphasized that prioritizing genes for marker-assisted selection and genome editing requires consideration of the specificity of their regulatory effects, since selection for a single target trait may influence other agronomically important characteristics. The analysis showed that genes and proteins regulating salinity tolerance are associated with 67 other phenotypic traits, including biomass, leaf area, grain morphology, and others, which reflects pleiotropic effects.

Three genes – OsPIL13, Ehd1, and OsGA2Ox3 – are positive regulators of both salinity tolerance and such agronomically important traits as grain quality, seed dormancy period, and grain length. This makes them promising candidates for breeding and genome editing, since their modulation may simultaneously increase salt tolerance and improve grain quality. At the same time, the genes OsWRKY63, OsRAM2, and OsABF4 enhance rice tolerance to salinity but are associated with negative regulation of seed dormancy period, grain protein content, and plant resistance to Fusarium graminearum and F. pseudograminearum, which must be considered in breeding programs.

According to SmartCrop, 21 genes are involved exclusively in the regulation of salinity tolerance and are not associated with the regulation of agronomically important traits or resistance to pathogens, which makes them suitable candidates for targeted breeding aimed at increasing salt tolerance.

**Fig. 6.**
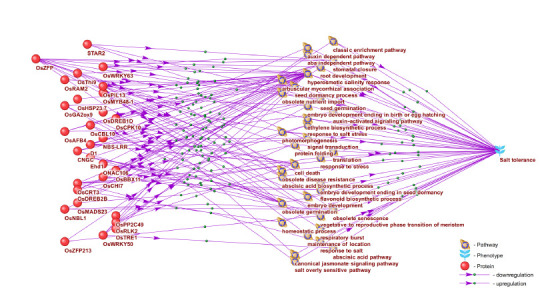
Regulatory relationships between genes associated with rice salinity tolerance and biological pathways involved in the regulation of this trait.

Another important factor that must be taken into account when selecting genes for marker-assisted selection and/ or genome editing is the potential bidirectionality of their regulatory effects, since gene products may either stimulate or suppress biological processes involved in the positive or negative regulation of the target trait. To assess such bidirectionality, the “Pathway Wizard” module of the ANDSystem program was used to identify regulatory relationships between the protein products of the 30 genes associated with rice salinity tolerance and the biological processes that, in turn, participate in regulating this trait (Fig. 6).

Among the genes regulating salinity tolerance, particular interest is drawn to OsMYB48-1, OsCPK10, OsCBL10, OsDREB2B, OsRAM2, and NBS-LRR, which exhibit a unidirectional effect in the form of positive regulation of key processes that ensure tolerance to salt stress (hyperosmotic salinity response, stomatal closure, ABA-independent pathway, etc.). The high degree of connectivity of these genes with the target trait, combined with the unidirectional nature of their regulatory action, suggests that their use in markerassisted selection or genome editing may have a more direct and pronounced impact on increasing salt tolerance compared with other candidates.

## Conclusion

The SmartCrop knowledge base is a specialized version of the ANDSystem software information platform, adapted for the tasks of rice and wheat genetics and breeding. It integrates information on a wide range of entities – genes, proteins, metabolites, noncoding RNAs, biological processes, breeding-relevant and phenotypic traits, pathogens, as well as biotic and abiotic factors – and their relationships. This architecture provides extensive opportunities for studying the molecular genetic mechanisms of plant stress tolerance, as well as for selecting genes, genetic markers, and phenotypic traits within the framework of marker-assisted selection of crop plants

Examples of SmartCrop applications for the functional annotation of differentially expressed genes in bread wheat under salt stress and for planning experiments to increase rice salinity tolerance using marker-assisted selection have demonstrated the high efficiency of the system and its potential for solving applied problems in breeding and genome editing.

## Conflict of interest

The authors declare no conflict of interest.
